# APOBEC3A/B Polymorphism Is Not Associated with Human Papillomavirus Infection and Cervical Carcinogenesis

**DOI:** 10.3390/pathogens12050636

**Published:** 2023-04-23

**Authors:** Eliza Pizarro Castilha, Rafaela Roberta de Jaime Curti, Janaina Nicolau de Oliveira, Glauco Akelinghton Freire Vitiello, Roberta Losi Guembarovski, José d’Oliveira Couto-Filho, Karen Brajão de Oliveira

**Affiliations:** 1 Department of Pathological Sciences, Biological Sciences Center, State University of Londrina, Londrina 86057-970, PR, Brazil; 2 Department of Biological Sciences, Biological Sciences Center, State University of Londrina, Londrina 86057-970, PR, Brazil; 3 Londrina Cancer Hospital, Londrina 86015-520, PR, Brazil

**Keywords:** *Human papillomavirus*, deaminases APOBEC, mutation accumulation

## Abstract

The persistence of a high-risk *Human papillomavirus* (HPV-HR) infection of the cervix results in different manifestations of lesions depending on the immunologic capacity of the host. Variations in apolipoprotein B mRNA editing enzyme catalytic polypeptide (APOBEC)-like genes, such as the APOBEC3A/B deletion hybrid polymorphism (A3A/B), may contribute to cervical malignancy in the presence of HPV. The aim of this study was to investigate the association between the A3A/B polymorphism and HPV infection and the development of cervical intraepithelial lesions and cervical cancer in Brazilian women. The study enrolled 369 women, who were categorized according to the presence of infection and subdivided according to the degree of intraepithelial lesion and cervical cancer. APOBEC3A/B was genotyped by allele-specific polymerase chain reaction (PCR). As for the A3A/B polymorphism, the distribution of genotypes was similar between groups and among the analyzed subgroups. There were no significant differences in the presence of infection or development of lesions, even after exclusion of confounding factors. This is the first study to show that the A3A/B polymorphism is not associated with HPV infection and the development of intraepithelial lesions and cervical cancer in Brazilian women.

## 1. Introduction

A *Human papillomavirus* (HPV) infection of the cervix results in different outcomes depending on the type of virus, persistence of infection, and intrinsic immune properties of the host [1]. Asymptomatic infections or infections that result in benign lesions are more common than the malignant lesions that are precursors to cervical cancer [2]. However, this virus is necessary to cause cervical cancer [3].

Variations in the apolipoprotein B mRNA editing enzyme catalytic polypeptide 3 (APOBEC3)-like gene, such as the APOBEC3A and APOBEC3B deletion hybrid (A3A/B), may be associated with persistent viral infections and the establishment of the somatic mutations that predispose to cellular malignancies [4].

The variant A3A/B allele is characterized by the deletion of 29,936 base pairs (bp) between the fifth exon of A3A and the eighth exon of A3B [5]. It results in a hybrid gene encoding a wild-type A3A-identical transcript fused to the 3’ untranslated region (UTR) of A3B [4,6,7]. As a result, exclusion of the A3B protein occurs, and posttranscriptional regulation of A3A is reduced, increasing the nucleocytoplasmic levels of the protein [4]. The A3A/B hybrid transcript is 10 to 20 times more stable and more highly expressed than the wild-type transcript [8].

Increased expression of the A3A protein encoded by the wild-type allele and/or the variant allele favors the formation of homodimers that tend to interact with specific sites (5’TC) in RNA or single-stranded DNA (ssDNA) [9,10]. The most important consequence is the deamination of cytidines (C) to uridines (U) [11], which generates double-strand breaks (DSB) or somatic mutations through transversions (C > G) or transitions (C > T) in the absence or failure of DNA repair [12]. These A3A signature mutations are found in bladder cancer [13], cervical cancer [4,14], breast cancer [15,16], ovarian cancer [17], head and neck cancer [18], and lung cancer [19].

In cervical cancer, the variant A3A/B allele amplifies the somatic mutational burden caused by the virus, confirms the genomic instability of cervical cells, and increases viral diversity, leading to neoplastic changes in the cervix [12,19]. Considering that HPV infections occur worldwide [20] and that cervical cancer is the third-most-common cancer in Brazilian women [21], the study of possible sources of somatic mutations, such as the A3A/B polymorphism, is essential for understanding the mechanisms of the malignancy of cervical cell lesions.

The aim of this study was to investigate the association between the A3A/B polymorphism and HPV infection, the development of cervical intraepithelial lesions, and cervical cancer.

## 2. Materials and Methods

### 2.1. Samples Characterization

The State University of Londrina’s Institutional Human Ethics Committee in Paraná, Brazil, approved this study (CEP/UEL 133/2012; CAAE 05505912.0.0000.5231). Informed consent was obtained from all participants, and they were informed about the study’s purpose and procedures. Women who participated in this case-control study were recruited from public health services in Londrina-PR, Brazil. Samples were collected from a nonrandomly selected biobank of 700 women (obtained by convenience). A structured questionnaire was completed to gather sociodemographic, reproductive, and sexual behavioral data. Participants were categorized based on the presence or absence of HPV DNA, and cervical cytology results were obtained from medical records.

Cervical cell samples were collected using cytobrushes from cervicovaginal smear, and peripheral blood was obtained through venipuncture using anticoagulant tubes. Genomic DNA was extracted from cervical brushings using DNAzol (InvitrogenTM Inc., Carlsbad, CA, USA) and from peripheral blood using the Biopur Mini Spin Plus Kit (Biometrix^®^, Curitiba, PR, Brazil). The DNA concentration was assessed using a NanoDrop 2000c TM spectrophotometer (Thermo Fisher Scientific, Walthan, MA, USA), and purity was determined by the absorbance ratio at 260 nm and 280 nm.

### 2.2. Human Papillomavirus Detection

The detection of HPV was performed using the MY09 (5′-CGTCCMAARGGAWACTGATC-3′) and MY11 (5′-GCMCAGGGWCATAA-YAATGG-3′) primers, which were specifically designed to amplify a conserved region of 450 base-pairs (bp) within the L1 gene of HPV [22]. To ensure the validity of the results, the human β-globin gene (268 bp fragment) was co-amplified using the GH20 (5′-GAAGAGCCAAGGACAGGTAC-3′) and PC04 (5′-CAACTTCATCCACGTTCACC-3′) primers under the same HPV PCR conditions [23]. Controls without DNA and HeLa cells with HPV18 integrated DNA were used as negative and positive controls, respectively. The PCR products were visualized by electrophoresis on a 10% polyacrylamide gel and stained with silver nitrate.

### 2.3. APOBEC3A/B Polymorphism Genotyping

Differentiation between alleles for the APOBEC3A/B polymorphism was performed by allele-specific PCR technique [6], using specific primers for the variant allele (del) (forward: 5′-TAGGTGCCACCCCGAT-3′ e reverse 5′-TTGAGCATAATCTTACTCTTGTAC-3′) and for the wild allele (ins) (forward 5′-TTGGTGCTGCCCCCTC-3′ e reverse 5′-TAGAGACTGAGGCCCAT-3′). Primers for the variant allele sequence generate a 700 bp PCR product after variant allele-specific amplification with the deletion. Primers for the wild allele amplify only the insertion configuration and generate 490 bp products. In addition, each of the samples that appeared to be homozygous for the variant allele was genotyped with a second set of oligonucleotides for the wild allele (ins2: forward 5′-TGTCCCTTTTCAGAGTTTGAGTA-3′ and reverse 5′-TGGAGCCAATTAATCACTTCAT-3′), generating a 705 bp amplification product.

### 2.4. Statistical Analysis

This study analyzed a population divided into two groups based on their HPV infection status: uninfected and infected. Infected patients were further categorized based on the severity of cervical lesions observed (without cervical lesion, with low-grade intraepithelial lesion (LSIL), high-grade intraepithelial lesion (HSIL), and cervical cancer) according to the result of cytological examination. The study then used the chi-square test or Fisher’s exact test, as appropriate, to analyze the relationship between socio-epidemiological variables and the infection, lesion, or cancer groups, and employed logistic regression to predict the influence of studied polymorphisms on the development of infections and lesions. Tests were conducted using SPSS statistics 22.0 software (SPSS Inc., Chicago, IL, USA), with a significance level of *p* < 0.05.

## 3. Results

### 3.1. Characteristics of the Study Population

The study population consisted of 369 women: 207 (61.1%) were positive for HPV DNA, and 162 (38.9%) were negative. The infected (HPV-positive) women were divided into groups with no lesions (*n* = 74), low-grade squamous intraepithelial lesions (LSIL) (*n* = 14), high-grade squamous intraepithelial lesions (HSIL) (*n* = 45), and cervical cancer (*n* = 69).

Sociodemographic data, sexual behavior, and reproductive data are shown in Table 1 and Table 2, respectively. HPV infection was more common in women aged ≤24 and ≥55 years (*p* = 0.003), smokers (*p* = 0.009), with a monthly income ≤ 1 minimum wage (*p* = 0.028), who used contraceptives (*p* = 0.001) and preservatives (*p* = 0.01), and who had their first sexual intercourse at age 17 years or younger (*p* = 0.03).

### 3.2. Association between Squamous Intraepithelial Lesions and Cancer in the HPV-Positive Samples and Sociodemographic, Sexual Behavioral, and Reproductive Data

In the association of squamous intraepithelial lesion extent with sociodemographic characteristics, a significant association was observed for age (≥55 years, *p* < 0.001), smoking (ex-smoker, *p* = 0.032), educational status (incomplete primary education, *p* = 0.019), and monthly income (*p* = 0.013), as shown in Table 3. The association between squamous lesions and sexual behavioral and reproductive characteristics was observed in patients who used contraceptives (*p* < 0.001) and did not use preservatives (*p* = 0.032), as shown in Table 4.

### 3.3. Distribution of APOBEC3A/B Polymorphism between HPV Infection, LSIL, HSIL, and Cervical Cancer

**Figure 1:**
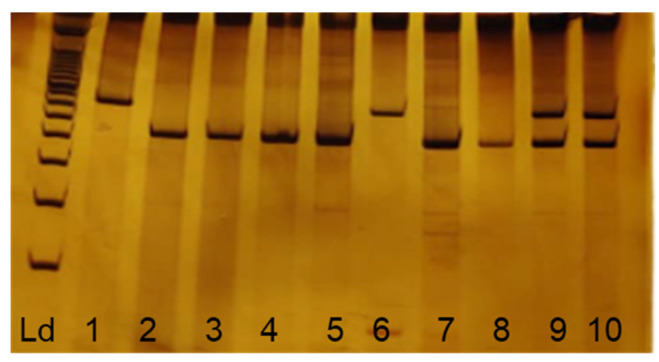
Electrophoretic profile of APOBEC3A/B polymorphisms. Ld: 100 bp molecular standard; 2, 3, 4, 5, 7, and 8 homozygous for wild-type allele (490 bp); 9 and 10 heterozygous (490 bp and 700 bp) and 1 and 6 homozygous for variant allele (700 bp).

Patients were divided into three groups according to the genotype for the polymorphism: homozygous for the wild-type allele (ins/ins), heterozygous (ins/del), and homozygous for the variant allele (del/del). The electrophoretic profile for each of these variants is shown in Figure 1. Of the total 369 women selected for the study, 242 (65.6%) were homozygous for the wild type, 103 (27.9%) were heterozygous, and 24 (6.5%) were homozygous for the variant allele.

According to the data in Table 5, it was found that in the HPV-uninfected group, the homozygous wild-type genotype was present in 103 women (63.8%), the heterozygous in 51 women (31.4%), and the variant homozygous in 8 women (4.8%). In the HPV-infected group, 139 (67.1%) women were homozygous for the wild-type allele, 52 (25.2%) were heterozygous, and 16 (7.7%) were homozygous for the variant genotypes. No significant difference was found between the HPV-uninfected and HPV-infected groups (*p* = 0.275), and there was also no significant association in multivariate logistic regression adjusting for age, smoking status, monthly income, contraceptives, preservatives, age at sexarche, and sexual partners.

When the group of HPV-infected patients was analyzed according to the degree of intraepithelial lesion, categorized as No Lesion, LSIL, HSIL, and Cancer, no significant association (*p* = 0.361) was found with polymorphism (Table 6).

When analyzed by multivariate logistic regression, no significant association was found between lesions’ grades and A3A/B polymorphism, even after adjusting for potential confounding factors (contraceptives, preservatives, age, smoking status, and monthly income) (Table 7).

## 4. Discussion

In this study, we investigated for the first time in a Brazilian sample the association between A3A/B deletion and HPV infection and the development of intraepithelial lesions and cervical cancer. We also confirmed the description of the sociodemographic, sexual, and reproductive aspects associated with a greater likelihood of persistent HPV infection and the development of low-grade (LSIL) and high-grade (HSIL) squamous intraepithelial lesions and cervical cancer in the same population.

A higher frequency of patients without lesions or with LSIL than with HSIL or cervical cancer was expected in the infected group, when we considered the probability of the progression of HPV lesions [2]. However, in our work, the proportion of women with HSIL and cervical cancer was higher than that of LSIL, possibly because the samples were from public health services. Brazilian epidemiological data show that 70% of diagnoses in women treated by public health services occur at an advanced stage of the disease, resulting in a worse prognosis [24], which confirms the frequency found here.

The characteristics that were significantly associated with a higher frequency of HPV infection and the development of intraepithelial lesions in the studied group were age, smoking, monthly income, contraceptives use, preservatives use, age of first sexual intercourse, and educational stage.

Age is a characteristic of susceptibility to HPV infection and lesion development that has been well-established in previous studies. Women younger than 24 years or older than 55 years are more affected by infection, as shown in this study [25,26,27,28,29]. Younger women have more intense sexual activity, which favors transmission of oncogenic HPV types [25]. The early onset of sexual activity, sexarche, also favors infection because the immature epithelium is more exposed to the virus [30]. At the other end of the scale is women 55 years of age and older, who become susceptible to HPV infection because of increased sexual activity due to increased life expectancy and quality of life [31] and possible reactivation of latent viruses due to hormonal changes at the menopause [26]. The persistence of HPV infection, immune response limited by aging or preexisting syndromes/diseases, and accumulation of mutations due to cellular senescence partially justify the increase in intraepithelial lesions and cervical cancer in a manner proportional to age [1,32,33].

We have shown here that smoking is related to cancer development and HPV infection, confirming previous studies by our group [27,29,34]. This association may be due to the fact that the DNA damage, immunosuppression, and epithelial dysplasia caused by cigarette components directly affect tumor development [35].

Regarding contraceptives use, we found a significant association with HPV infection and cervical cancer development, which may be due to endometrial hyperplasia caused by hormonal imbalance, as noted in a recent meta-analysis [36].

The use of preservatives reduces transmission and favors viral elimination [37]. We hypothesize that the association of infection with the use of preservatives is due to the environment in which these patients are, i.e., possibly because these women are treated in a primary health care unit and have already received instructions for preservatives use. However, consistent with expectations, 80.8% of those with HPV did not use preservatives, and 80.1% of them developed significantly more severe lesions.

The association between ethnicity and susceptibility to HPV infection is complex, especially since the data were obtained based on patient self-report. The Brazilian population is highly admixed, with heterogeneous ancestry that includes both indigenous people and immigrants from Europe, Africa, and Asia [38]. Moreover, race, as well as access to education and health services, is a determinant of socioeconomic and cultural advancement in Brazilian society. Therefore, we divided the individuals into Caucasians and non-Caucasians to not only obtain an unbiased view of the data but also take into account the social differences between these groups. Considering that Brazil is a continental country and the distribution of this heritage varies by region, it is advisable to point out that the study group is from the same region and represents a sample from the southern region, colonized mainly by European immigrants [39].

The overexpression or variation of APOBEC3 genes has been cited as a possible source of aberrant DNA editing during replication, repair, or transcription, which is capable of inducing somatic mutations that cause genomic instability in cancer cells [40]. Data from The Cancer Genome Atlas (TCGA) show the presence of signature mutations (C-to-T transitions or C-to-G transversions in TCX sequences, where the underlined base is the mutated base, and X can be any base) [41] attributed to APOBEC3A (A3A) and APOBEC3B (A3B) activity in breast, bladder, lung, head and neck, and cervical cancers [4,42].

The A3A/B polymorphism confers a greater susceptibility to cancer by generating a hybrid transcript between A3A and A3B 3′UTR that is more stable and more highly expressed in cells [17]. A3A signature mutations are more common in cancer cells [43], and the A3A protein is able to hypermutate nuclear DNA and generate double-stranded DNA breaks (DSBs), cause apoptosis, and promote mutations in cancer-causing genes, contributing to tumorigenesis [8].

In cervical cancer, there is a microenvironment that favors the mutagenic activity of A3A: 1. The accumulation of A3A is stimulated by the production of interferons in response to viral infection [42]; 2. the direct action of the oncoprotein E7 of HPV-HR inhibits the proteassomal degradation of A3A, making it more stable [44]; and 3. the replication stress promoted by HPV-HR increases cellular ssDNA exposure and makes them susceptible to A3 restriction [43].

In our study, we found no association of A3A/B polymorphism with infection, LSIL, HSIL, or cervical cancer, which is consistent with previous studies that failed to detect this association in other sample groups [15]; however, this result is in contrast to another study that showed this association [44].

The absence of the association of the variant allele with cancer found in our study reflects the population in southern Brazil. This result is consistent with a previous breast cancer study conducted with an independent sample from the same geographic region [45]. The frequency of polymorphisms depends on the ancestry of the sample group. The frequency of A3A/B varies among Asian (37%), Native American (57.7%), European (6%), and African (0.9%) populations [6,13,17]. Here, as mentioned previously, the study group consisted predominantly of self-identified Caucasian women with probable European ancestry, so the prevalence of this genetic variant is considerably lower. The low frequency of the variant allele in studies with independent samples may be explained by the Caucasian characteristics of the region, with the variant allele being more common in Asian populations.

Nevertheless, there is no consensus in the literature on the role of A3A and A3B in the development of lesions and cervical cancer [46], highlighting the need for studies such as ours to investigate this association. A3B is considered a major protein responsible for mutagenesis in cancer, which may explain the lack of association of the polymorphism (lack of translated A3B region) with carcinogenesis [16]. In contrast, other studies cite A3A as the source of somatic mutations in the host genome and confirm a significant association of the polymorphism with cervical lesions [11,47]. This inconsistency in defining the A3 mechanism in cervical cancer makes it difficult to interpret the role of the polymorphism in this situation.

Regarding the study limitations, sampling by convenience may have an influence on the homogeneity of the sample characteristics, mainly the sociodemographic variables. Moreover, the experiment design does not include longitudinal monitoring of the infection and lesion development in the participants, which means that a causal relation of the statistically significant variables is not possible to be assumed. Finally, the specific detection of high- and low-risk infection could be helpful to better characterize the frequency and association of the polymorphism between the groups with lesions or cancer in this population. Despite the limitations that cannot be ignored, the importance of this work is given by the discussion of a genetic variation that is so rarely studied in the different branches of research. The non-association found does not invalidate the valuable discussion about a group of genes intrinsically related to the antiviral response and provides support for future basic research.

## 5. Conclusions

In this study, we examined the main sociodemographic characteristics and sexual and reproductive behaviors associated with HPV infection and the development of precursor lesions and cervical cancer. We showed that the APOBEC3A/B polymorphism does not contribute to HPV infection or to the development of low- or high-grade cervical intraepithelial lesions and cervical cancer in a southern Brazilian population. Finally, we would like to emphasize that more studies with ethnically different populations must be developed, in order to overcome the limitations described here and validate the non-association of this polymorphism with HPV infection and the resulting lesions.

## Figures and Tables

**Table 1:** Sociodemographic characteristics of HPV-positive patients and controls.

Variables		HPV−		HPV+		*p* Value *
		N	%	N	%	
Age (years)	≤24	10	6.2	26 *	12.6	**0.003**
	25–34	41	25.3	46	22.2	
	35–44	39	24.1	48	23.2	
	45–54	47	29.0	33	15.9	
	≥55	25	15.4	54 *	26.1	
Ethnicity	Caucasian	80	50.3	102	50.0	0.953
	Not Caucasian	79	49.7	102	50.0	
Smoking status	No	123	76.4	130	62.8	**0.009**
	Yes	22	13.7	54 *	26.1	
	Ex-smoker	16	9.9	23	11.1	
Educational stage ^b^	Incomplete elementary	48	30.2	85 *	41.7	0.082
	Complete elementary	18	11.3	27	13.2	
	Incomplete secondary	19	12.0	23	11.3	
	Complete secondary	56	35.2	57	28.0	
	Incomplete higher education	5	3.1	6	2.9	
	Complete higher education	13	8.2	6	2.9	
Marital status	Married	118	72.8	135	65.5	0.067
	Single	14	8.6	37 *	18.0	
	Divorced	22	13.6	22	10.7	
	Widowed	8	5.0	12	5.8	
Monthly income ^a^	≤1 min wage	70	44.3	75	54.3 *	**0.028**
	>1–3 min wages	80	50.6	54	39.1	
	>3–5 min wages	4	2.5	9	6.6	
	>5–7 min wages	3	2.0	0	0.0	
	≥7 min wages	1	0.6	0	0.0	

^a^ Based on Brazilian minimum wage (approximately USD 215.00). ^b^ Based on Brazilian educational system. * Analysis by two-sided chi-square (*Χ*^2^) test or Fisher’s test, when appropriate, and *p* < 0.05 set as significance level (SPSS Inc., Chicago, IL, USA). Some categories did not complete the total of patients due to lack of data. Bold values represent statistical significance (*p* < 0.05).

**Table 2:** Sexual behavioral and reproductive features of HPV-positive patients and controls.

Variables		HPV−		HPV+		*p* Value *
		N	%	N	%	
Contraceptives	No	115	71.9	114	55.3	**0.001**
	Yes	45	28.1	92 *	44.7	
Preservatives	No	143	90.5	164	80.8	**0.010**
	Yes	15	9.5	39 *	19.2	
Age at menarche (years)	≤11	38	23.6	32	23.0	0.509
	12	35	21.7	40	28.8	
	13	36	22.4	30	21.6	
	≥14	2	32.3	37	26.6	
Age at sexarche (years)	≤17	80	49.4	86 *	61.9	**0.030**
	≥18	82	50.6	53	38.1	
Sexual partners during their lifetime	1	64	40.0	36	26.0	0.072
	2	27	16.9	20	14.5	
	3	21	13.0	23	16.7	
	4	14	8.8	16	11.6	
	≥5	34	21.3	43	31.2	
Knowledge about HPV	No	28	17.5	37	26.6	0.102
	Have ever heard of HPV	88	55.0	74	53.2	
	Yes	44	27.5	28	20.2	
Knowledge about the transmission of HPV	No	69	43.1	63	45.7	0.661
	Yes	91	56.9	75	54.3	

* Analysis by two-sided chi-square (*Χ*^2^) test, and *p* < 0.05 set as significance level (SPSS Inc., Chicago, IL, USA). Some categories did not complete the total of patients due to lack of data. Bold values represent statistical significance (*p* < 0.05).

**Table 3:** Association between squamous intraepithelial lesions and cancer in the HPV-positive samples and sociodemographic characteristics.

Variables		HPV+								
		No Lesions		LSIL		HSIL		Cancer		*p* Value *
		N	%	N	%	N	%	N	%	
Age (years)	≤24	13	17.6	4	28.6	7	15.6	0	0.0	**<0.001**
	25–34	22	29.7	4	28.6	15	33.3	5	7.2	
	35–44	17	23.0	2	14.3	11	24.4	17	24.6	
	45–54	9	12.1	2	14.3	7	15.6	15	21.7	
	≥55	13	17.6	2	14.2	5	11.1	32 *	46.5	
Ethnicity	Caucasian	37	50.0	5	38.5	21	48.8	36	52.2	0.840
	Not Caucasian	37	50.0	8	61.5	22	51.2	33	47.8	
Smoking status	No	54	73.0	7	50.0	26	57.8	39	56.6	**0.032**
	Yes	16	21.6	6	42.9	16	35.6	15	21.7	
	Ex-smoker	4	5.4	1	7.1	3	6.6	15 *	21.7	
Educational stage ^b^	Incomplete elementary	20	27.0	5	35.7	18	41.9	40 *	58.8	**0.019**
	Complete elementary	9	12.2	1	7.1	5	11.6	11	16.2	
	Incomplete secondary	10	13.5	2	14.3	7	16.3	4	5.9	
	Complete secondary	30	40.5	6	42.9	9	20.9	10	14.7	
	Incomplete higher education	3	4.1	0	0.0	3	7.0	0	0.0	
	Complete higher education	2	2.7	0	0.0	1	2.3	3	4.4	
Marital status	Married	48	64.9	9	64.3	31	68.9	43	63.3	0.661
	Single	17	23.0	2	14.3	9	20.0	9	13.2	
	Divorced	5	6.8	2	14.3	5	11.1	9	13.2	
	Widowed	4	5.3	1	7.1	0	0.0	7	10.3	
Monthly income ^a^	≤1 min wage	31	41.9	8	57.1	30 *	71.4	3	100.0	**0.013**
	>1–3 min wages	34	45.9	6	42.9	12	28.6	0	0.0	
	>3–5 min wages	9	12.2	0	0.0	0	0.0	0	0.0	
	>5–7 min wages	0	0.0	0	0.0	0	0.0	0	0.0	
	≥7 min wages	0	0.0	0	0.0	0	0.0	0	0.0	

^a^ Based on Brazilian minimum wage (approximately USD 215.00). ^b^ Based on Brazilian educational system. * Analysis by two-sided chi-square (*Χ*^2^) test or Fisher’s test, when appropriate, and *p* < 0.05 set as significance level (SPSS Inc., Chicago, IL, USA). LSIL (low-grade squamous intraepithelial lesions); HSIL (high-grade squamous intraepithelial lesions); some categories did not complete the total of patients due to lack of data. Bold values represent statistical significance (*p* < 0.05).

**Table 4:** Association between squamous intraepithelial lesions and cancer in the HPV-positive samples and sexual behavioral and reproductive features.

Variables		HPV+								
		No Lesions		LSIL		HSIL		Cancer		*p* Value *
		N	%	N	%	N	%	N	%	
Contraceptives	No	53	71.6	6	42.9	27	60.0	25	36.8	**<0.001**
	Yes	21	28.4	8	57.1	18	40.0	43 *	63.2	
Preservatives	No	64	86.5	11	78.6	37	88.1 *	47	69.1	**0.032**
	Yes	10	13.5	3	21.4	5	11.9	21	30.9	
Age at menarche (years)	≤11	13	17.6	4	28.6	14	31.8	0	0.0	0.388
	12	25	33.8	2	14.2	11	25.0	2	66.7	
	13	14	18.9	4	28.6	11	25.0	0	0.0	
	≥14	22	29.7	4	28.6	8	18.2	1	33.3	
Age at sexarche (years)	≤17	41	55.4	10	71.4	31	70.5	3	100.0	0.167
	≥18	33	44.6	4	28.6	13	29.5	0	0.0	
Sexual partners during their lifetime	1	26	35.6	3	21.4	5	11.6	0	0.0	0.062
	2	10	13.7	3	21.4	4	9.3	0	0.0	
	3	10	13.7	4	28.6	9	20.9	0	0.0	
	4	6	8.2	3	21.4	6	14.0	1	33.3	
	≥5	21	28.8	1	7.2	19	44.2	2	66.7	
Knowledge about HPV	No	20	27.0	2	14.3	13	30.2	1	33.3	0.823
	Have ever heard of HPV	38	51.4	10	71.4	21	48.8	1	33.3	
	Yes	16	21.6	2	14.3	9	21.0	1	33.4	
Knowledge about the transmission of HPV	No	27	37.0	7	50.0	22	51.2	2	66.7	0.365
	Yes	46	63.0	7	50.0	21	48.8	1	33.3	

* Analysis by two-sided chi-square (*Χ*^2^) test or Fisher’s test, when appropriate, and *p* < 0.05 set as significance level (SPSS Inc., Chicago, IL, USA). LSIL (low-grade squamous intraepithelial lesions); HSIL (high-grade squamous intraepithelial lesions); some categories did not complete the total of patients due to lack of data. Bold values represent statistical significance (*p* < 0.05).

**Table 5:** Association between the polymorphism APOBEC3A/B and HPV infection by multinomial logistic regression.

Variables		HPV−	HPV+	*p* *	OR	CI	*p*	OR_adj_	CI_adj_	*p* **_adj_
		N (%)	N (%)							
Genotypic	ins/Ins	103 (63.8)	139 (67.1)	0.275	1	1	1	1	1	1
	ins/del	51 (31.4)	52 (25.2)		0.756	0.476–1.200	0.235	0.556	0.308–1.001	0.050
	del/del	8 (4.8)	16 (7.7)		1.482	0.611–3.595	0.384	1.647	0.539–5.036	0.382

* Analysis by two-sided chi-square (*Χ*^2^) test, and *p* < 0.05 set as significance level. OR (odds ratio) and CI (confidence interval) 95% estimated by multinomial logistic regression with “ins/ins” as reference and **_adj_ adjusted by age, smoking status, monthly income, contraceptives, preservatives, age at sexarche, and sexual partners (SPSS Inc., Chicago, IL, USA).

**Table 6:** Association between the polymorphism APOBEC3A/B, HPV infection and squamous intraepithelial lesions and cancer in the HPV-positive samples.

Variables		HPV+				*p* Value *
		No Lesions	LSIL	HSIL	Cancer	
		N (%)	N (%)	N (%)	N (%)	
Genotypic	ins/Ins	47 (63.5)	12 (85.8)	29 (64.4)	49 (71.0)	0.361
	ins/del	19 (25.7)	1 (7.1)	12 (26.7)	18 (26.1)	
	del/del	8 (10.8)	1 (7.1)	4 (8.9)	2 (2.9)	

* Analysis by two-sided Fisher’s exact test, and *p* < 0.05 set as significance level (SPSS Inc., Chicago, IL, USA). LSIL (low-grade squamous intraepithelial lesions); HSIL (high-grade squamous intraepithelial lesions); some categories did not complete the total of patients due to lack of data.

**Table 7:** Association between the polymorphism APOBEC3A/B and squamous intraepithelial lesions and cancer in the HPV-positive samples by multinomial logistic regression.

Variables		HPV+								
		LSIL			HSIL			Cancer		
		OR	CI	*p* *	OR	CI	*p* *	OR	CI	*p* *
Genotypic	ins/Ins	1	1	ref	1	1	1	1	1	1
	ins/del	0.206	0.025–1.698	0.142	1.024	0.434–2.415	0.958	0.909	0.425–1.941	0.805
	del/del	0.490	0.056–4.302	0.520	0.810	0.224–2.933	0.749	0.240	0.048–1.188	0.080
		OR_adj_	CI_adj_	*p* **_adj_	OR_adj_	CI_adj_	*p* **_adj_	OR_adj_	CI_adj_	*p* **_adj_
Genotypic	ins/Ins	1	1	ref	1	1	1	1	1	1
	ins/del	0.214	0.024–1.891	0.166	1.112	0.426–2.902	0.828	4.871–9	-	0.998
	del/del	0.489	0.047–5.127	0.551	0.559	0.100–3.141	0.509	2.230	0.085–58.29	0.630

* OR (odds ratio) and CI (confidence interval) 95% estimated by multinomial logistic regression with “ins/ins” as reference and **_adj_ adjusted by contraceptives, preservatives, age, smoking status, and monthly income (SPSS Inc., Chicago, IL, USA). LSIL (low-grade squamous intraepithelial lesions); HSIL (high-grade squamous intraepithelial lesions). Statistical significance (*p* < 0.05).

## Data Availability

Not applicable.
